# Electromyographic Assessment of Functional Symmetry of Paraspinal Muscles during Static Exercises in Adolescents with Idiopathic Scoliosis

**DOI:** 10.1155/2014/573276

**Published:** 2014-09-02

**Authors:** Wiesław Chwała, Agnieszka Koziana, Tadeusz Kasperczyk, Robert Walaszek, Maciej Płaszewski

**Affiliations:** ^1^Section of Biomechanics of the University School of Physical Education in Kraków, Al. Jana Pawła II 78, 31-571 Kraków, Poland; ^2^Institute of Physiotherapy of the Podhale State Higher Vocational School in Nowy Targ, ul. Kokoszków 71, 34-400 Nowy Targ, Poland; ^3^Section of Physiotherapy and Biological Regeneration of the University School of Physical Education in Kraków, Al. Jana Pawła II 78, 31-571 Kraków, Poland; ^4^Section of Biological Regeneration of the University School of Physical Education in Kraków, Al. Jana Pawła II 78, 31-871 Kraków, Poland; ^5^Institute of Physiotherapy, Faculty of Physical Education and Sport in Biała Podlaska, The Józef Piłsudski University School of Physical Education, Warszawa, ul. Akademicka 2, 21-500 Biała Podlaska, Poland

## Abstract

*Background*. The question of how to correct and rehabilitate scoliosis remains one of the most difficult problems of orthopaedics. Controversies continue to arise regarding various types of both symmetric and asymmetric scoliosis-specific therapeutic exercises. *Objective*. The aim of the present paper was to conduct an electromyographic assessment of functional symmetry of paraspinal muscles during symmetric and asymmetric exercises in adolescents with idiopathic scoliosis. *Materials and Methods*. The study was conducted in a group of 82 girls, mean age 12.4 ± 2.3 years with single- or double-major-idiopathic scoliosis, Cobb angle 24 ± 9.4°. The functional biopotentials during isometric work of paraspinal muscles in “at rest” position and during two symmetric and four asymmetric exercises were measured with the use of the Muscle Tester ME 6000 electromyograph. *Results*. In general, asymmetric exercises were characterised by larger differences in bioelectrical activity of paraspinal muscles, in comparison with symmetric exercises, both in the groups of patients with single-curve and double-curve scoliosis. *Conclusion*. During symmetric and asymmetric exercises, muscle tension patterns differed significantly in both groups, in comparison with the examination at rest, in most cases generating positive corrective patterns. Asymmetric exercises generated divergent muscle tension patterns on the convex and concave sides of the deformity.

## 1. Introduction

One of the commonly noted phenomena in the progress of idiopathic scoliosis (AIS) is the asymmetry of paraspinal muscle tension, with a predominance of biopotential on one side of the spinal curvature [1, 2]. This phenomenon has a significant impact on the mechanical conditions of the functioning of paraspinal muscles exposed to asymmetric loads. It can potentially lead to a shift in the relative position of proximal and distal muscle endings or in the length of muscle moments of force and their operating angle, thus influencing the value of muscle moments achieved in relation to the rotating axis of vertebral joints on the convex and concave sides of the curvature.

Reports are available which, on the basis of histological examinations, indicate a decrease in number of slow twitch muscle fibres on the concave side of the spinal curvature [3]. The reduction of bioelectric activity of muscles on the concave side of the curvature eventually leads to an impairment of their activity and obliteration of the proportion of slow twitch fibres due to inactivity. This is usually accompanied by the increase in the share of connective tissue in muscular structures, which considerably reduces their flexibility, thus rendering self-correction even more difficult. It is, however, difficult to accept the assumption [4] that therapeutic action aimed at strengthening of the concave side muscles can reduce the progression of scoliosis. From the point of view of biomechanics of human movement, in case of existing spinal deformity, any attempt at strengthening the concave side muscles is disadvantageous, as it impacts the curve of the spinal curvature just as the bow-string does the bow: the stronger it is strung, the more profound the curve becomes [5].

It should be noted here that the predominance in the bioelectric tension of the paraspinal muscles at the convex side is a natural defence mechanism of muscles and should be considered a positive corrective mechanism against the progression of scoliosis [5, 6].

In view of the above it was assumed that an increase of the predominance of the bioelectrical activity on the convex side of the spinal curvature during symmetric and asymmetric exercises in comparison with the bioelectrical activity pattern measured in the resting position would indicate a positive impact of those exercises on the progression of scoliosis, while a disadvantageous corrective pattern would be characterized by an increased predominance of bioelectrical activity of the paraspinal muscles on the concave side.

## 2. Objective

The aim of the present study was to assess the impact of symmetric and asymmetric exercises on the bioelectrical activity of the paraspinal muscles and symmetry of their functioning in comparison with the rest tension pattern in girls with idiopathic scoliosis.

The following research questions were formulated.

1. Do the employed symmetric and asymmetric exercises considerably alter the bioelectrical activity pattern of prevertebral muscles in comparison with the values in resting position?
2. Which of the symmetric and asymmetric exercises generate positive corrective pattern of bioelectrical activity of paraspinal muscles on the apex of spinal curvature in subjects with single- and double-curve scoliosis?
3. Do the symmetric and asymmetric exercises have the same impact on single- and double-curve scoliosis?

## 3. Materials and Methods

The overall electromyographic examinations were conducted in girls (*n* = 82) with single- and double major idiopathic scoliosis (44 and 38 girls, resp.), Cobb angle between 13 and 35 degrees in the single-curve, and between 12 and 53 degrees in the double-curve group, as measured on the standing A-P radiograph. The subjects were divided into two groups. The subjects' characteristics are presented in Table 1.

The participants were recruited from three rehabilitation centres located in southern Poland, which in their therapeutic process employed both the symmetric and asymmetric exercises, with the mobilization of the physiological curves of the spine and active three-plane self-correction.

The exclusion criteria were the history of spinal surgery, brace therapy, neurological diseases, or acute back pain.

The study has received ethical approval from the Scientific Research Ethics Committee (Bioethics Committee attached to the Chamber of Physicians in Cracow; expertise no. 23/KBL/OIL/2007 of March 21, 2007). Furthermore, parents of the recruited girls consented in a written form to the subjects' participation in the project.

The functional biopotentials during isometric exercises were measured with the Muscle Tester ME 6000 electromyograph (Mega Electronics Ltd.), with the frequency of 1000 Hz. The electrodes were located on the subjects' skin at the apex of the curvature on both sides of the spine. Measurements were taken in accordance with the ISEK and SENIAM standards [7]. The obtained electromyographic record was filtered with the use of the high-pass filter with the cut-off frequency of 30 Hz. In the computer analysis, the variable of the area beneath the integrated electromyogram chart was used [5] according to the following equation:

(1) $$\begin{array}{c} A_{i}=\overset{t}{\underset{to}{\int }}U_{i}\left(t\right)dt, \end{array}$$

where *A*_*i*_ is the area under the chart of the RMS-averaged functional biopotential of paraspinal muscles within the interval of 15 seconds [*μ*Vs], *U*_*i*_(*t*) is the value of the integrated functional biopotential of paraspinal muscles [*μ*V], and *t* is the duration of the analyzed functional biopotential of paraspinal muscles (15 s).

Statistical analysis was then applied to the quotients of the above values as measured on both the convex and concave sides of the curvature, which were called the ISBAM coefficient (*Index of Symmetry of Bioelectrical Activity of Muscles*) and calculated according to the following equation:

(2) $$\begin{array}{c} \text{ISBAM}=\frac{A_{i}\left(\text{convex}\right)}{A_{i}\left(\text{concave}\right)}, \end{array}$$

where ISBAM is the index of symmetry of bioelectrical activity of muscles calculated as the quotient of the area under the iEMG curve on the convex and concave side; *A*_*i*_(convex) is the value of the analyzed area under the curve of the integrated iEMG of convex side paraspinal muscles within the interval of 15 s [*μ*Vs]; *A*_*i*_(concave) is the value of the analyzed area under the curve of the integrated iEMG of concave side paraspinal muscles within the interval of 15 s [*μ*Vs].

The measurements of functional biopotentials of paraspinal muscles were taken in the resting position and during six (two symmetric and four asymmetric) exercises.

**Figure 1:**
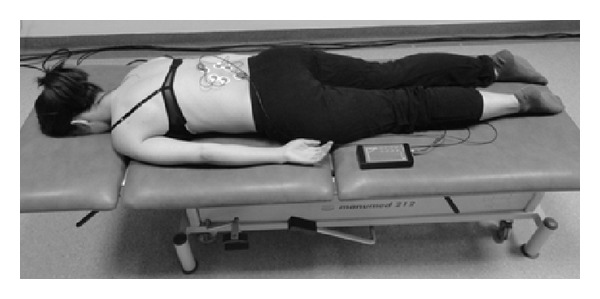
Measurement position at rest (*S*).

**Figure 2:**
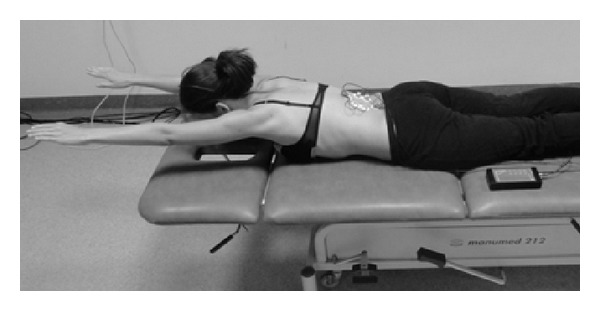
Measurement position during the *S*_1_ exercise.

**Figure 3:**
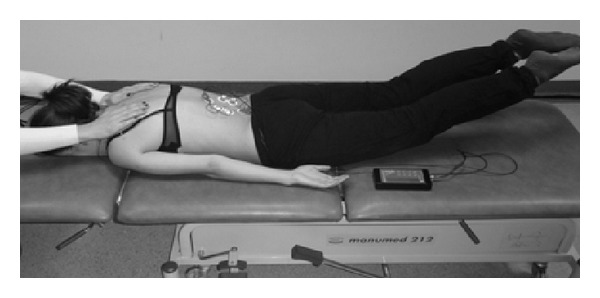
Measurement position during the *S*_2_ exercise.

**Figure 4:**
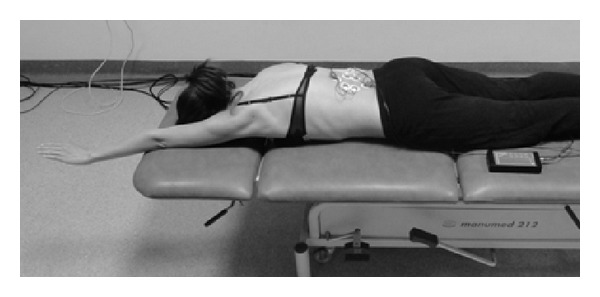
Measurement position during the *A*_1_ exercise.

**Figure 5:**
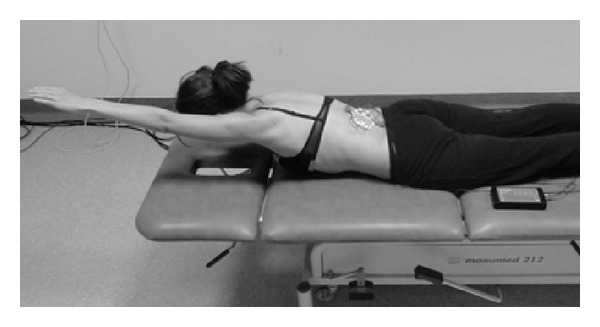
Measurement position during the *A*_2_ exercise.

**Figure 6:**
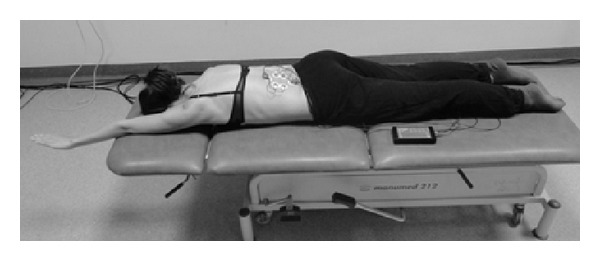
Measurement position during the *A*_3_ exercise, single-curve scoliosis.

**Figure 7:**
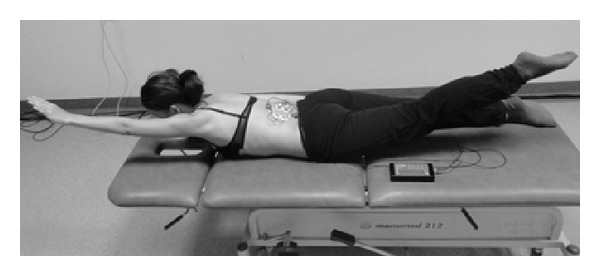
Measurement position during the *A*_4_ exercise, single-curve scoliosis.

1. Resting position (*S*), initial position: the subject lies prone with arms along the trunk, with the body in resting position and the muscles relaxed (Figure 1).
2. Symmetric exercise (*S*_1_), initial position: the subject lies prone with both the upper extremities extended and moved forward and ankles stabilised vertically to the settee. In C-shaped and S-shaped scoliosis: the subject is to raise her legs symmetrically and sustain a static position (Figure 2).
3. Symmetric exercise (*S*_2_), initial position: the subject lies prone with both the upper extremities along the trunk and shoulder girdle stabilised. In C-shaped and S-shaped scoliosis: the subject is to raise her legs symmetrically and sustain a static position (Figure 3).
4. Asymmetric load-free exercise (*A*_1_), initial position: the subject lies prone with the convex side upper limb extended along the trunk and the concave side upper limb moved cranially and extended in the elbow joint and in the radiocarpal joint. The subject is to move cranially the concave side upper limb and hold it in a static position (Figure 4).
5. Asymmetric loaded exercise (*A*_2_), initial position: the subject lies prone with the convex side upper limb extended along the trunk and the concave side upper limb moved cranially and extended in the elbow joint and in the radiocarpal joint. The subject is to raise her trunk together with the concave side upper limb moved cranially (the limb as an extension of the trunk) and hold her body in a static position (Figure 5).
6. Asymmetric load-free exercise (*A*_3_), initial position: the subject lies prone with the convex side upper limb along the trunk and the concave side upper limb moved cranially and extended in the elbow joint and in the radiocarpal joint. Single-curve scoliosis: the subject is to move cranially the concave side upper limb and, at the same time, push caudally the concave side lower limb (Figure 6). Double-curve scoliosis: the subject is to move cranially the arm on the upper curvature's concave side and, simultaneously, push caudally the lower limb on the concave side of the lower spinal curvature. In both cases the subject is to hold her body in a static position.
7. Asymmetric loaded exercise (*A*_4_), initial position: the subject lies prone with the convex side arm along the trunk and the concave side arm moved cranially and extended in the elbow joint and in the radiocarpal joint. Single-curve scoliosis: the subject is to push cranially and raise her arm on the concave side and, simultaneously, raise and push caudally the concave side lower limb (Figure 7). Double-curve scoliosis: the subject is to push cranially and raise the arm on the upper concave side of the deformity and, simultaneously, raise and push caudally the leg on the concave side of the distal curve. In both cases the subject is to hold her body in a static position.

## 4. Statistics

The STATISTICA 6.0 package was used in the statistical analysis. The distribution of the examined variables was assessed with the Shapiro-Wilk test for normal distribution. The significance of differences between the mean values of the analyzed variables was assessed with the McNemar's chi² test for dependent variables.

## 5. Results

Table 2 presents the mean values (±*s*) of the ISBAM symmetry coefficient and the results of the test of statistical significance of differences between the symmetry coefficient as calculated in the resting position (*S*) and during individual symmetric and asymmetric exercises in both groups.

## 6. Discussion

Researchers differ in their opinions regarding therapeutic management following the diagnosis of idiopathic scoliosis. It is widely assumed that scoliosis should not be treated conservatively but instead periodically observed and, in case of considerable progression to the threshold values of the curvature angle, surgical intervention is suggested [8].

On the other hand, contradictory opinions are formulated by Maruyama et al. [9] and by Negrini et al. [10], who recommend the soonest possible application of physical exercises (PES) aimed at correcting or reducing the progression of scoliosis.

Different conservative therapeutic approaches are used; among them, PES play significant role. Some studies stress the practical effects of such an attempt to the problem of scoliosis [11, 12].

Fusco et al. [13] rated the effectiveness of PES at level 1b according to the Oxford Centre for Evidence-Based Medicine.

In PES-based therapeutic methods, asymmetric corrective exercises dominate. They form the basis of self-corrective exercises through which patients attempt to correct the positioning of the vertebral column and other segments of the spine in an intended direction—in all the three planes—and to maintain this improved positioning during their everyday activities [14–18]. Some therapies employ symmetric mobilizing exercises aimed at improving of spinal flexibility in the sagittal plane and correct formation of the lumbar lordosis and thoracic kyphosis [19].

Another example of exerting an asymmetric influence without self-correction is the use of the MedX Rotary Torso Machine in the therapy of scoliosis [20, 21].

An example of using symmetric exercises based on the principle of autoelongation without self-correction (the Milwaukee method) in the therapy of scoliosis is presented by Stone et al. [22]. However, the claimed positive influence of exercises based on this method upon the correction of scoliosis has not been supported with evidence. In spite of the multiplicity of conservative methods of treatment of scoliosis, their effectiveness still remains unproven [23, 24].

Chwala et al. [6] report that the loading of the postural muscles with symmetric moments of weights, occurring during everyday activities, can, in individual, distinct cases of spine deformities, both lead to the progression of scoliosis and have a corrective influence, which also depends on the patient's habitual posture alignments. Similar opinions are presented by Gram and Hasan [25].

Based on the presented findings, it can be assumed that the mean value of the ISBAM coefficient during the “at rest” examination (*S*) in persons with single curve scoliosis was close to one. In the remaining exercises the mean value of the same factor differed significantly from the value recorded at rest (*P* < 0.05), therefore indicating a significant predominant involvement of the convex side muscles. The exception was the asymmetric exercise with a load (*A*_2_), where the recorded muscle tension pattern was similar to that observed in the resting position. In the same exercise, a similar number of positive and negative muscular activity patterns occurred (Table 3).

The highest bioelectrical activity of the convex side paraspinal muscles was observed in the (*A*_1_) asymmetric load-free exercise, consisting of actively stretching the concave side of the curve (in more than 80% of cases, a positive corrective pattern was recorded). Significant and beneficial differences of corrective patterns in relation to the resting position were also found in both types of symmetric exercises (*P* < 0.05), where the proportion of positive patterns of bioelectrical muscle activity was close to 80%.

The mean value of the muscle tension index as measured in the resting position (*S*) in persons with double-curve scoliosis did not differ significantly between the proximal and distal curves and suggested a stronger involvement of the concave side muscles. In accordance to that, 84% and 66% of negative patterns of bioelectrical activity were recorded from the thoracic and lumbar curves, respectively.

During symmetric exercises (*S*_1_ and *S*_2_), statistically significant predominance of bioelectrical activity of the convex over the concave side muscles were found, both at the thoracic and lumbar curves (*P* < 0.01 and *P* < 0.001, resp.). Those manifested through higher values of the ISBAM coefficient, in comparison with the resting recordings. Positive pattern of bioelectrical activity of paraspinal muscles was observed in about 70% of subjects.

A similar functional pattern of paraspinal muscles was measured during the *A*_3_ asymmetric exercise, which consisted in actively stretching the concave side of the thoracic and lumbar spinal curvatures. Namely, a marked predominance of the convex side muscles was observed, as measured at both thoracic and lumbar curves. In the same exercise, the mean value of the ISBAM symmetry coefficient differed significantly in relation to the resting values (*P* < 0.001). In the majority of subjects, a positive corrective pattern occurred at the level of the apex of both curves.

The *A*_2_ and *A*_4_ asymmetric exercises generated very similar patterns of paraspinal muscle tension, with predominance of the convex side muscles at the apex of the lumbar curve (above 70% of positive corrective patterns), and an opposite tendency was observed at the apex of the thoracic curve (negative corrective patterns occurred in almost all of the subjects). Chwała et al. [5] observed similar tendencies in a group of adults with AIS, treated in adolescence with scoliosis-specific exercises. The mean ISBAM values recorded at both curves differed significantly in relation to the resting activity pattern of the paraspinal muscles (*P* < 0.05 and *P* < 0.001 for thoracic and lumbar curves, resp.).

The *A*_1_ asymmetric load-free exercise was characterized by a reversed pattern of muscle activity, in comparison with the *A*_2_ and *A*_4_ exercises. Significant increase of electric biopotential was found on the convex side of the thoracic curve, in comparison with the pattern recorded at rest (*P* < 0.001). The predominance of bioelectrical activity of concave side paraspinal muscles increased significantly at the lumbar curve (*P* < 0.05), which resulted in decrease of the ISBAM values. During this exercise, a positive corrective pattern at the thoracic curve was observed in 82% of the subjects, while in 89% of the participants the exercise manifested with a negative corrective pattern at the lumbar level.

Taking into consideration that each individual scoliosis manifests with a unique set of factors determining its biomechanical development, the principle of individualization should be followed in the selection of exercises. In order to obtain a proper corrective pattern, such a selection should be supervised by a highly trained professional, based on EMG examination, enabling an objective choice of exercises, adapting the selection to the patient's individual characteristics of muscle activity.

Although distinct and significant tendencies to change the muscle activity patterns were observed in individual symmetric and asymmetric exercises, in relation to the patterns recorded at rest, it should be noted that, in some subjects from both the single- and double-major-curve groups, reversed patterns of muscle activity were observed. The observed differences may have resulted from the variability of individual performance technique of the subsequent exercises. During the therapeutic process, patients use their own motor habits and, until their performance becomes automatic, their individual attempts are highly variable. This also implies the risk of different impacts of the same exercises in individual patients, arising from the specific character of each case of scoliosis and individual patient's motor experience, as well as from the quality of patient's performance and repeatability of the corrective patterns of the exercises. Consequently, individualized, case-specific approach to the therapy, including planning and supervising the therapeutic process of adolescents with idiopathic scoliosis, is highly recommended. Otherwise, in individual cases, the same set of exercises may be ineffective or may lead to unexpected or opposite responses. Furthermore, individualised approach to initial diagnostics and periodic examination during the process of therapy can be helpful in increasing the effectiveness of the selected physical exercises or of the self-correction activities of patients during the therapy and in daily life. Such approach, as indicated by Fusco et al. [13], is among key conditions for success of the therapy of scoliosis.

## 7. Conclusions

1. In single-curve scoliosis, the most beneficial corrective factor was recorded during the performance of both the asymmetric load-free exercises (*A*_1_ and *A*_3_) and during the symmetric exercises (*S*_1_ and *S*_2_).
2. The patterns of muscle activity during symmetric exercises in the groups of subjects with single curve and double major curve scoliosis differed significantly from the resting values, generating in the majority of the participants a positive corrective factor.
3. In the majority of the subjects with double major curve scoliosis, contrary muscle tension patterns were observed at the apexes of the curves during asymmetric exercises with a load.
4. In consideration of the different patterns of bioelectrical activity of paraspinal muscles recorded during the same exercises in both groups, the principle of individualised selection of exercises to be applied in the therapy should be observed, with the help of the analysis of bioelectrical activity of paraspinal muscles.
5. As various patterns of bioelectrical activity of paraspinal muscles were measured in both groups during the same exercises, it can be assumed that the exercises should be applied according to the principle of individualised selection of exercises, facilitated by the application of the analysis of bioelectrical activity of paraspinal musculature.

## Figures and Tables

**Table 1:** Characteristics of the subjects.

Variables	Single curves *n* = 44	Double major curves *n* = 38
Age (years)	12.2 ± 2.47	12.8 ± 2.16
Body mass (kg)	42.2 ± 12.2	47.82 ± 10.9
Height (m)	1.52 ± 0.14	1.59 ± 0.12
Curve angle (Cobb) (°)	14.07 ± 3.28	L-25 ± 11.7 Th-24 ± 12.03

**Table 2:** Average values (±*s*) of the ISBAM symmetry coefficient and results of the test of statistical significance (McNemar's chi² test) of differences between the symmetry coefficient as measured “at rest” and symmetry coefficient as measured during individual symmetric and asymmetric exercises in subjects with C-shaped and S-shaped idiopathic scoliosis.

PES	ISBAM AIS C-shaped curves *N* = 44	ISBAM AIS S-shaped curves (upper curve Th) n = 38	ISBAM AIS S-shaped curves (lower curve L) n = 38
*S*	1.04 ± 0.60	0.85 ± 0.42	0.60 ± 0.34
*S*_1_	1.19 ± 0.27∗	1.34 ± 0.52∗∗∗	1.20 ± 0.39∗∗∗
*S*_2_	1.26 ± 0.39∗	1.20 ± 0.65∗∗	1.20 ± 0.42∗∗∗
*A*_1_	2.33 ± 1.32∗∗∗	1.76 ± 0.88∗∗∗	0.46 ± 0.26∗
*A*_2_	1.03 ± 0.36	0.48 ± 0.29∗∗∗	1.12 ± 0.40∗∗∗
*A*_3_	1.96 ± 0.94∗∗∗	1.80 ± 0.93∗∗∗	1.39 ± 0.97∗∗∗
*A*_4_	1.29 ± 0.47∗∗	0.57 ± 0.41∗	1.52 ± 0.56∗∗∗

PES: physical exercises.

*P < 0.05, ∗∗*P* < 0.01, ∗∗∗*P* < 0.001.

**Table 3:** Relative share of positive and negative patterns of bioelectrical activity of paraspinal muscles in individual exercises in persons with C-shaped and S-shaped scoliosis.

Type of scoliosis	*S*		*S*_1_		*S*_2_		*A*_1_		*A*_2_		*A*_3_		*A*_4_	
	[%]													
	P	N	P	N	P	N	P	N	P	N	P	N	P	N
AIS C-shaped curves *n* = 44	43	57	79	21	77	23	82	18	59	41	84	16	77	13

AIS S-shaped curves (upper curve-Th) *n* = 38	34	66	74	26	68	32	82	18	1	99	76	24	11	89

AIS S-shaped curves (lower curve-L) *n* = 38	16	84	71	29	76	24	11	89	73	27	68	32	84	16

P: positive pattern of bioelectrical activity of muscles; N: negative pattern of bioelectrical activity of muscles.
